# Different pathological response and histological features following neoadjuvant chemotherapy or chemo-immunotherapy in resected non-small cell lung cancer

**DOI:** 10.3389/fonc.2023.1115156

**Published:** 2023-02-09

**Authors:** Greta Alì, Anello Marcello Poma, Iosè Di Stefano, Carmelina Cristina Zirafa, Alessandra Lenzini, Giulia Martinelli, Gaetano Romano, Antonio Chella, Editta Baldini, Franca Melfi, Gabriella Fontanini

**Affiliations:** ^1^ Unit of Pathological Anatomy, University Hospital of Pisa, Pisa, Italy; ^2^ Department of Surgical, Medical, Molecular Pathology and Critical Area, University of Pisa, Pisa, Italy; ^3^ Multispecialty Centre for Surgery, Minimally Invasive and Robotic Thoracic Surgery, University Hospital of Pisa, Pisa, Italy; ^4^ Unit of Pneumology, University Hospital of Pisa, Pisa, Italy; ^5^ Medical Oncology, San Luca Hospital, Lucca, Italy

**Keywords:** non-small cell lung cancer, neoadjuvant therapy, chemo-immunotherapy, pathological response, prognosis, biomarkers

## Abstract

**Introduction:**

Non-small cell lung cancer (NSCLC) is the leading cause of cancer incidence and mortality worldwide. Neoadjuvant chemo-immunotherapy has led to clinical benefits in resectable NSCLC in comparison to chemo-therapy alone. Major pathological response (MPR) and pathological complete response (pCR) have been used as surrogates of neoadjuvant therapy response and clinical outcomes. However, the factors affecting the pathological response are still controversial. Therefore, in this study we retrospectively examined MPR and pCR in two different cohorts of NSCLC patients, 14 treated by chemotherapy and 12 by chemo-immunotherapy in the neoadjuvant setting.

**Methods:**

In resected tumor specimens, different histological characteristics were evaluated: necrosis, fibrosis, inflammation, presence of organizing pneumonia, granuloma, cholesterol cleft, and reactive epithelial alterations. In addition, we evaluated how MPR impacts on event-free survival (EFS) and overall survival (OS). In a small group of patients treated by chemo-immunotherapy, a gene expression analysis of the Hippo pathway was performed both in preoperative biopsies and matched post-surgical specimens.

**Results:**

We observed a better pathological response in the chemo-immunotherapy treated cohort: 6/12 patients (50.0%) achieved a MPR ≤10% and 1/12 (8.3%) achieved pCR both on primary tumor and on lymph nodes. On the contrary, no patient treated with chemotherapy alone achieved pCR or MPR ≤10%. A higher amount of stroma in the neoplastic bed was observed in patients treated with immuno-chemotherapy. Moreover, patients achieving better MPR (including pCR) had significantly improved overall survival (OS) and event-free survival (EFS). After neoadjuvant chemo-immunotherapy, residual tumors showed a remarkable upregulation of genes consistent with the activation of YAP/TAZ. Also, alternative checkpoint, such as CTLA-4, were enhanced.

**Discussion:**

Our findings showed that neoadjuvant chemo-immunotherapy treatment improves MPR and pCR thus resulting in better EFS and OS. Moreover, a combined treatment could induce different morphological and molecular changes in comparison to chemotherapy alone, thus giving new insights in the assessment of pathological response.

## 1 Introduction

Non-small cell lung cancer (NSCLC) is the greatest cause of cancer death. Despite recent improvements in the treatment of advanced NSCLC, little is known about therapy efficacy in resectable tumors (1, 2). Although the advances in staging, surgical techniques, and the introduction of adjuvant chemotherapy for stage II and III NSCLC, a large number of operated patients experience disease recurrence (3). In particular, patients with resectable NSCLC at high risk of recurrence may benefit from neoadjuvant or adjuvant chemotherapy, but the 5-year overall survival is reached only in 5% of cases (4). In recent years, immunotherapy emerged as a therapeutic option for lung cancer, and neoadjuvant immunotherapy can be a good alternative for patients with resectable NSCLC. In the neoadjuvant setting immunotherapy, a combination of chemotherapy and immunotherapy, and targeted therapies are currently under investigation (5–7).

However, there are no established guidelines about the assessment of response to neoadjuvant therapy on resected lung cancer specimens. Over the years, different approaches have been used to assess pathological response, including pathological complete response (pCR) and major pathological response (MPR) (8, 9). Previous studies have suggested a positive association between pathological response, mainly pCR, and clinical outcome of patients. It has been demonstrated that patients with NSCLC showing a MPR of 10% or less have a significantly better outcome after neoadjuvant chemotherapy (10–12). As regards immunotherapy alone data are still limited.,. However, a neoadjuvant chemo-immunotherapy may increase the proportion of patients achieving a major pathological response (MPR) (13, 14). Indeed, available studies (15) showed that neoadjuvant chemo-immunotherapy can be more effective than chemotherapy alone in patients with resectable NSCLC. However, a considerable percentage of tumors do not completely respond to neoadjuvant chemo-immunotherapy, and patients may develop early disease progression (16). Thus, the identification of patients without a substantial pathological response is crucial to adjust treatment. To date, no studies have compared the pathological response to chemotherapy and to chemo-immunotherapy in NSCLC. In this study we retrospectively examined the efficacy of neoadjuvant chemotherapy and chemo-immunotherapy in patients with NSCLC comparing the MPR and several histological characteristics such as necrosis, fibrosis, and inflammation both in the tumor and in the collateral lung parenchyma. In addition, we evaluated how MPR impacts on event-free survival (EFS) and overall survival (OS). Moreover, in a small group of patients treated with chemo-immunotherapy, we performed a gene expression analysis of the Hippo pathway, crucial for tissue repair and associated with treatment resistance, both in preoperative biopsies and matched post-surgical specimens.

## 2 Materials and methods

### 2.1 Patient selection

We retrospectively enrolled 26 NSCLC patients, including 14 who had received chemotherapy and 12 who had received chemo-immunotherapy in the neoadjuvant setting, from April 2017 to January 2021 and from December 2018 to October 2021, respectively. In detail, Surgical specimens of patients were collected from the archives of the Operative Unit of Pathological Anatomy III of the University Hospital of Pisa. In detail, tumors included 14 adenocarcinoma (ADC), 8 squamous cell carcinoma (SCC), 1 adenosquamous carcinoma, 1 pleomorphic carcinoma, and 2 large cell neuroendocrine carcinoma obtained from patients who underwent surgical resection at the Department of Cardiothoracic Surgery of the University Hospital of Pisa. For 5 patients treated with combined chemo-immunotherapy, we also collected pre-surgical biopsies. Participation in this study required informed consent. Treatment regimens and indications for surgery were determined by a multidisciplinary team. All patients received surgery within 4-6 weeks after neoadjuvant chemo-immunotherapy or chemotherapy. In detail, 14 patients received 2-4 cycles of a conventional platinum-based doublet chemotherapy regimen, whereas 12 patients received 2-6 cycles of a conventional platinum-based doublet chemotherapy regimen combined with PD-1 (pembrolizumab, n=4) or PD-L1 inhibitors, (atezolizumab in 6 cases and durvalumab in 2 cases). As per standard institutional procedures, all surgical resections were performed with thoracotomy, video-assisted thoracoscopic surgery, or robotic-assisted pulmonary resection.

Clinical information including patient sex, age, molecular status, PD-L1 immunohistochemical expression, EFS, and OS, were reviewed for each patient. EFS was considered as the time from the start of neoadjuvant treatment until disease progression. OS was considered from the start of therapy to the date of death or censored at the last follow-up. This study was approved by the ethics committee “Comitato Etico di Area Vasta Nord Ovest” (CEAVNO) for Clinical Experimentation (Protocol Number: ID19211).

### 2.2 Pathological response evaluation

Information on neoadjuvant therapy, such as medication and course of treatment, was documented. Tumors were staged according to the American Joint Committee on Cancer Lung Cancer Staging, 8th edition (17). The pathological response was assessed independently by two pathologists (GA and IDS) that evaluated both pCR and MPR. MPR was defined as residual viable tumor cells in the primary tumor bed and sampled lymph nodes. MPR was reported both as a continuous variable and using the 10% cut-off, whereas pCR was defined as the complete absence of residual viable tumor cells in the primary tumor (8, 9).

In detail, according to the recommendations of The International Association for the Study of Lung Cancer (IASLC) (9), if the tumor bed was small (≤ 3 cm) the tumor was entirely sampled. If the tumor bed was larger than 3 cm, the tumor was cut in serial sections approximately 0.5 cm thick and after gross inspection the most representative cross section showing viable tumor was sampled (at least one cross section of the entire tumor). If no viable tumor was identified in the cross sections, the remaining tumor tissue was examined histologically to see if any viable tumor was present. Besides tumor cells, the percentages of major components of the tumor bed such as necrosis and stroma (which includes inflammatory cells and fibrosis) were calculated with the total adding up to 100%, as previously described (9). The percentage of residual viable tumor was estimated by comparing the estimated cross-sectional area of the viable tumor foci with estimated cross-sectional areas of necrosis, fibrosis, and inflammation on each hematoxylin and eosin slide. The results for all slides were averaged together to determine the mean values for each patient (10). For lymph node pathological response, the same approach was used for histological evaluation that was used for the resected lung cancer.

Moreover, we calculated the pathological regression (PR) evaluated as 100 - the percentage of residual viable tumor cells. Finally, we evaluated other histological features in the tumor microenvironment such as inflammation, fibrosis, presence of organizing pneumonia, granuloma, cholesterol cleft, and reactive epithelial alterations.

PD-L1 expression before treatment was detected by using the rabbit monoclonal primary antibody SP263 and the expression was evaluated by tumor proportion score (TPS).

### 2.3 Gene expression analysis

For all samples, tumor cell percentage was estimated independently by two expert pathologists and tumor component was enriched by manual macrodissection before nucleic acid extraction. In detail, total RNA was purified from three-to-four unstained formalin-fixed paraffin embedded (FFPE) sections (10 µm-thick) using the Qiagen RNeasy FFPE kit (Qiagen, Hilden, Germany), and according to the manufacturer’s suggestions. RNA quality and concentration were assessed using an Xpose spectrophotometer (Trinean, Gentbrugge, Belgium). About 150 ng of total RNA were used for gene expression analysis using the nCounter system (nanoString Technologies, Seattle, WA, USA). A custom panel of 88 genes was designed including 10 housekeeping genes (i.e., *CLTC*, *EDC3*, *GAPDH*, *GUSB*, *HPRT1*, *MRPS5*, *NUBP1*, *PGK1*, *PRPF38A*, *SF3A3*), 4 genes encoding for immune checkpoint proteins (i.e., *CD274*, *CTLA4*, *PDCD1*, *VSIR*) and 74 genes belonging to the Hippo pathway. The complete list of genes is reported in **Supplementary Table S1**. Total RNA was hybridized with capture and reporter probes at 60°C for 20 hours; cleanup of samples and counts of digital reports were performed as described by the manufacturer (nanoString Technologies).

### 2.4 Data analyses and statistics

Continuous variables are presented as median and interquartile range (IQR) and were tested by the Mann-Whitney test or by the Kruskal-Wallis test followed by the Dunn test. Categorical variables were tested by the Fisher exact test. Correlations between continuous variables were evaluated by Pearson correlation.

For gene expression analysis, raw counts were normalized using the nCounter Advanced Analysis (nanoString Technologies). Differentially expressed genes were computed following the procedures of the nCounter Advanced Analysis. *P*-values were adjusted by the Benjamini-Hochberg method, and a false discovery rate (FDR) of 0.05 was considered significant. Principal component analysis (PCA) was performed using PCAtools v.2.10.0 package, while hierarchical clustering was carried out using heatmap3 v. 1.1.9 package. Optimal cut-off for MPR was assessed by the Contal and O’Quigley’s method and using the survMisc v.0.5.6 package. Survival curves were estimated by the Kaplan-Meier method following the procedures of survival v.3.4-0 package and plotted using survminer v.0.4.9 package. Hazard ratio (HR) was estimated using the Cox regression method.

All analysis and plots were generated in R environment v.4.1.2 (https://www.r-project.org/, last accessed November 14, 2022) unless otherwise specified.

## 3 Results

### 3.1 Patient clinico-pathological characteristics and different treatment regimens

Clinico-pathological characteristics of patients are summarized in **Table 1**. Twenty-three patients with resectable NSCLC were included: 8 were females and 18 males aged from 41 to 78 years old (median age of 66 years). Twelve patients were treated with combined chemo-immunotherapy and 14 with chemotherapy. Most patients (50.8%) had stage IA to IB disease, 5 (19.2%) had stage IIB, 7 (26.2%) had stage IIIA and IIIB, and one (3.8%) patient had stage IVA. For the combined immuno-chemotherapy treated patients, 10 (83.3%) had a PD-L1 TPS of 1% or higher and 2 (16.7%) had TPS of 50% or higher. For chemotherapy treated patients, 3 (21.4%) had PD-L1 negative, 9 (64.2%) had tumor with low PD-L1 expression (TPS 1-49%), and 2 (14.4%) had tumor with high PD-L1 (TPS ≥ 50%).

**Table 1:** Clinicopathological characteristics and pathological response of NSCLC patients according to treatment regimens.

Features	All Patients (n = 26)	CIT Patients (n = 12)	CT Patients (n = 14)	P value
*Age (years), median (IQR)*	69 (57-71)	67 (58.5-70.5)	69 (59.5-71.75)	0.55
*Sex, n (%)* *female* *male*	8 (30.8) 18 (69.2)	3 (25) 9 (75)	5 (35.7) 9 (64.3)	0.43
*Size of tumor (cm)*, *median (IQR)*	4.35 (2.50-6.22)	4.35 (2.87-6.00)	4.15 (2.42-6.45)	0.88
*Histology, n (%)* *ADC* *SCC* *Others*	14 (53.8) 8 (30.8) 4 (15.4)	7 (58.3) 3 (25.0) 2 (16.7)	7 (50.0) 5 (35.6) 2 (14.4)	0.87
*pT, n (%)* *T1 (a+b+c)* *T2 (a+b)* *T3* *T4*	7 (26.2) 6 (23.1) 8 (30.8) 4 (15.4)	5 (41.8) 3 (25.0) 5 (41.8) 1 (8.3)	3 (21.4) 5 (35.7) 3 (21.4) 3 (21.4)	0.26
*pN, n (%)* *N0* *N1* *N2*	16 (61.5) 6 (23.1) 4 (15.4)	8 (66.6) 2 (16.7) 2 (16.7)	8 (57.2) 4 (28.6) 2 (14.3)	0.86
*Clinical Stage (8th edition), n (%)* *IA (IA1, IA2, IA3) - IB* *IIB* *IIIA - IIIB* *IVA*	13 (50.8) 5 (19.2) 7 (26.2) 1 (3.8)	6 (50) 2 (16.7) 4 (33.3) 0	7 (50.0) 3 (21.4) 3 (21.4) 1 (7.2)	0.82
*Grade, n (%)* *G2* *G3*	(n = 23) 9 (39.1) 14 (60.9)	(n = 11) 4 (36.4) 7 (63.6)	(n = 12) 5 (41.7) 7 (58.3)	0.67
*MPR, median (IQR)*	36.5 (9-59.5)	6 (2.75-34.75)	56 (12-72)	**0.001**
*MPR, n (%)* *≤ 10%* *> 10%*	7 (26.9) 19 (73.1)	7 (58.2) 5 (41.8)	0 14 (100)	**0.001**
*PD-L1 expression, n (%)* *Negative (< 1%)* *Positive (≥ 1% - 49%)* *(≥ 50%)*	3 (11.5) 19 (73.1) 4 (15.4)	0 11 (91.7) 1 (8.3)	3 (21.4) 8 (57.2) 3 (21.4)	0.20
*Mutational status, n (%)* *WT* *KRAS mutation* *RET rearrangement* *NA*	14 (53.9) 6 (23.1) 1 (3.8) 5 (19.2)	6 (50.0) 4 (33.4) 1 (8.3) 1 (8.3)	8 (49.9) 2 (14.4) 0 4 (35.7)	0.36

CIT, chemo-immunotherapy; CT, chemotherapy; IQR, interquartile range; ADC, adenocarcinoma; SCC, squamous cell carcinoma; Others comprise: 1 adenosquamous cell carcinoma; 1 pleomorphic carcinoma; and 2 large cell neuroendocrine carcinoma; WT, wild-type; NA, not available.

Bold *p*-value: value below 0.05 was considered significant.

No significant differences between the two neoadjuvant therapy groups were observed in terms of age, sex, histological tumor type, size of tumors, TNM stage, grade, PD-L1 expression, and mutational status (**Table 1**).

### 3.2 Pathological response and morphological data

**Figure 1:**
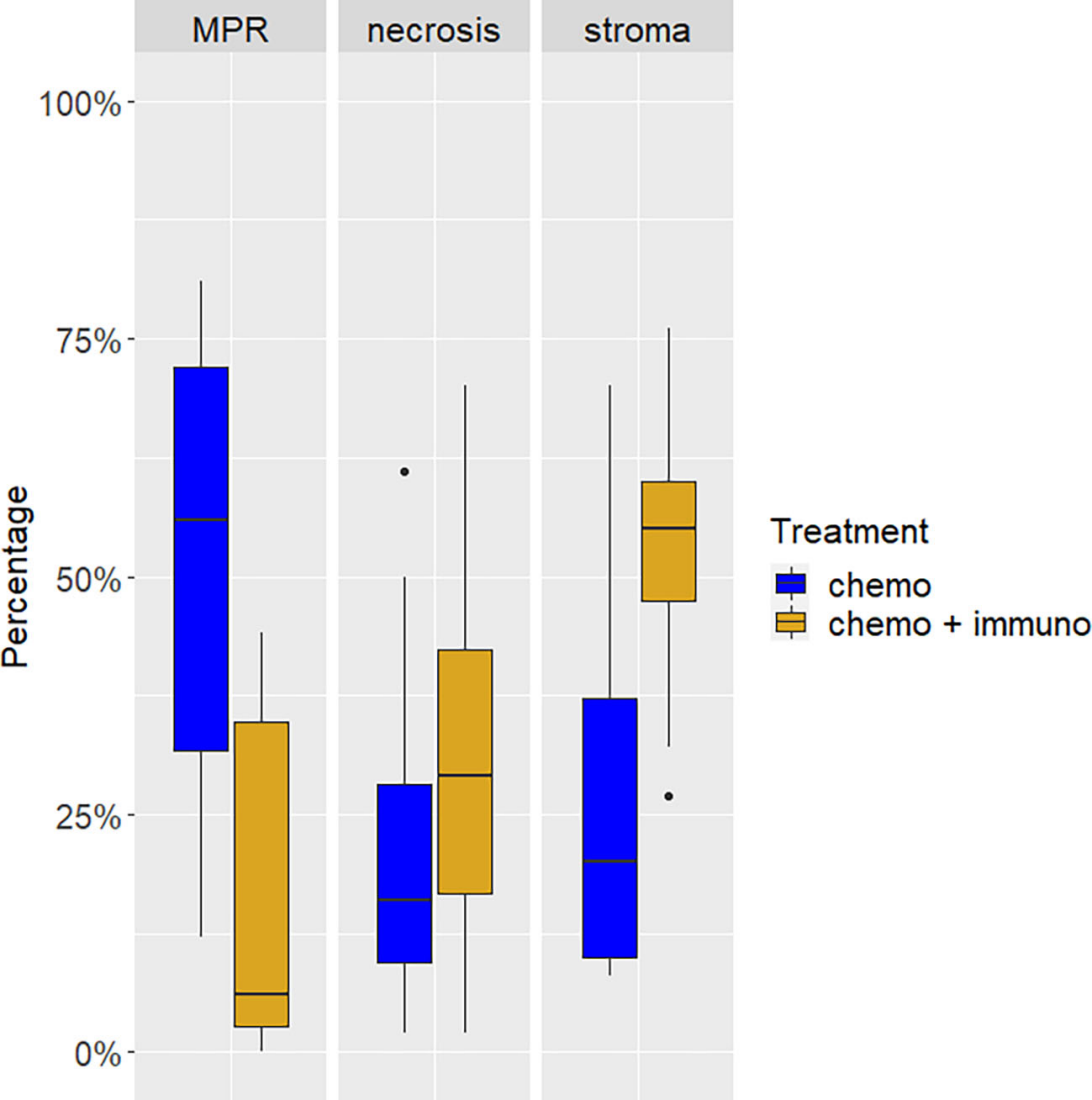
Tumor composition after neoadjuvant treatment with chemo- or chemo-immunotherapy. The proportion of viable tumor cells (major pathological response, MPR) was lower in tumors from patients of the chemo-immunotherapy cohort. These cases showed also a higher proportion of necrosis and stroma.

**Figure 2:**
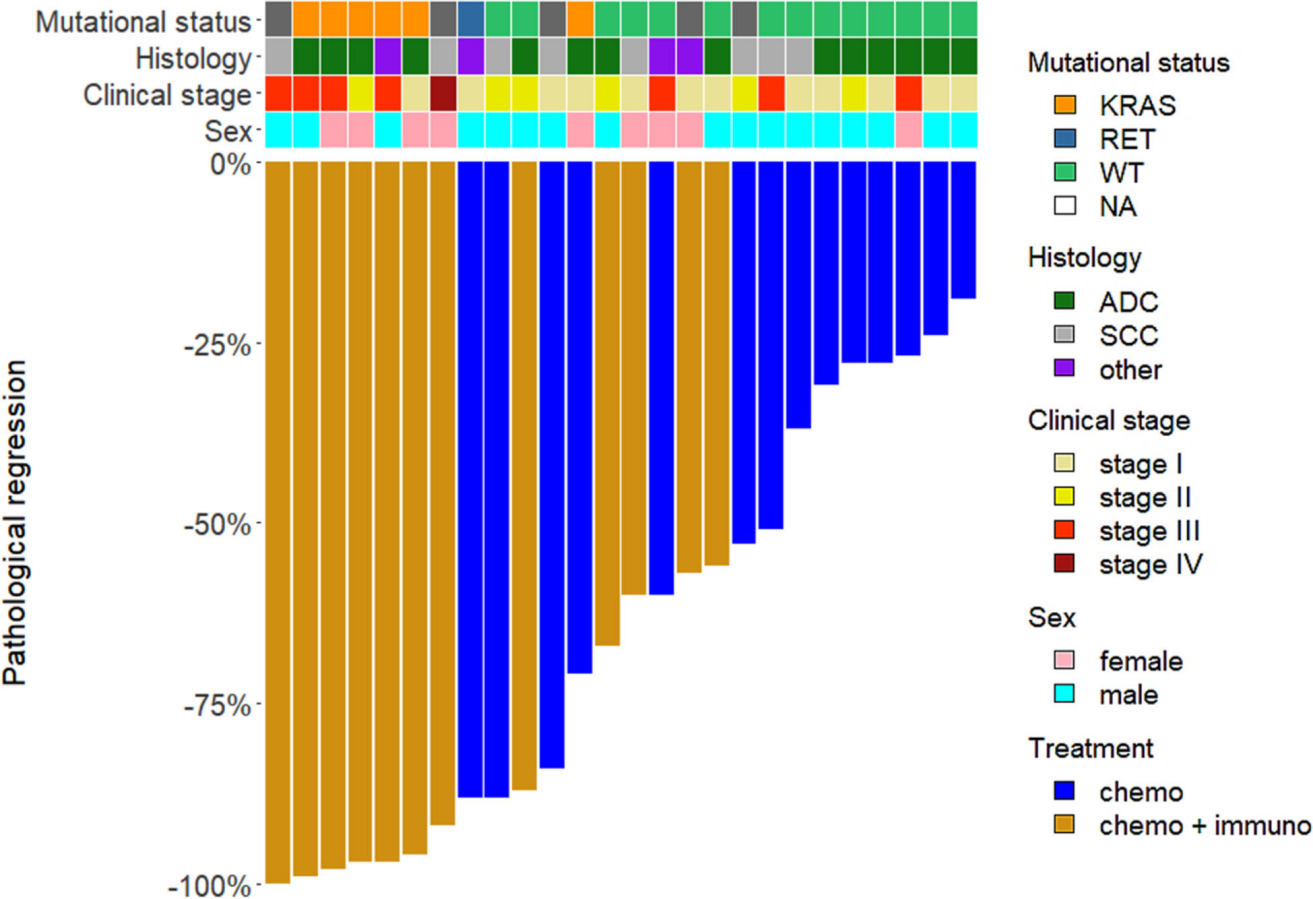
Waterfall plot of pathological response. The bars represent patients according to the different neoadjuvant treatments, chemotherapy alone (chemo) and chemo-immunotherapy (chemo-immuno). The upper rows show clinic-pathological characteristics of patients (mutational status, histology, clinical stage, sex). WT, wild-type; NA, not available; ADC, adenocarcinoma; SCC, squamous cell carcinoma.

Major pathological response was different between the two cohorts of patients both as continuous variable (*p* < 0.0001) and considering the 10% cut-off (*p* < 0.0001) (**Table 1**). In detail, 7 patients treated with combined immuno-chemotherapy (58.3%) achieved a MPR ≤10% and one patient (3.8%) pCR in the primary tumor and sampled lymph nodes. On the other hand, no chemotherapy treated patients achieved MPR ≤10% or pCR (**Figure 1**). The waterfall plot shows pathological regression in the resected primary lung tumor after neoadjuvant treatment (**Figure 2**).

**Figure 3:**
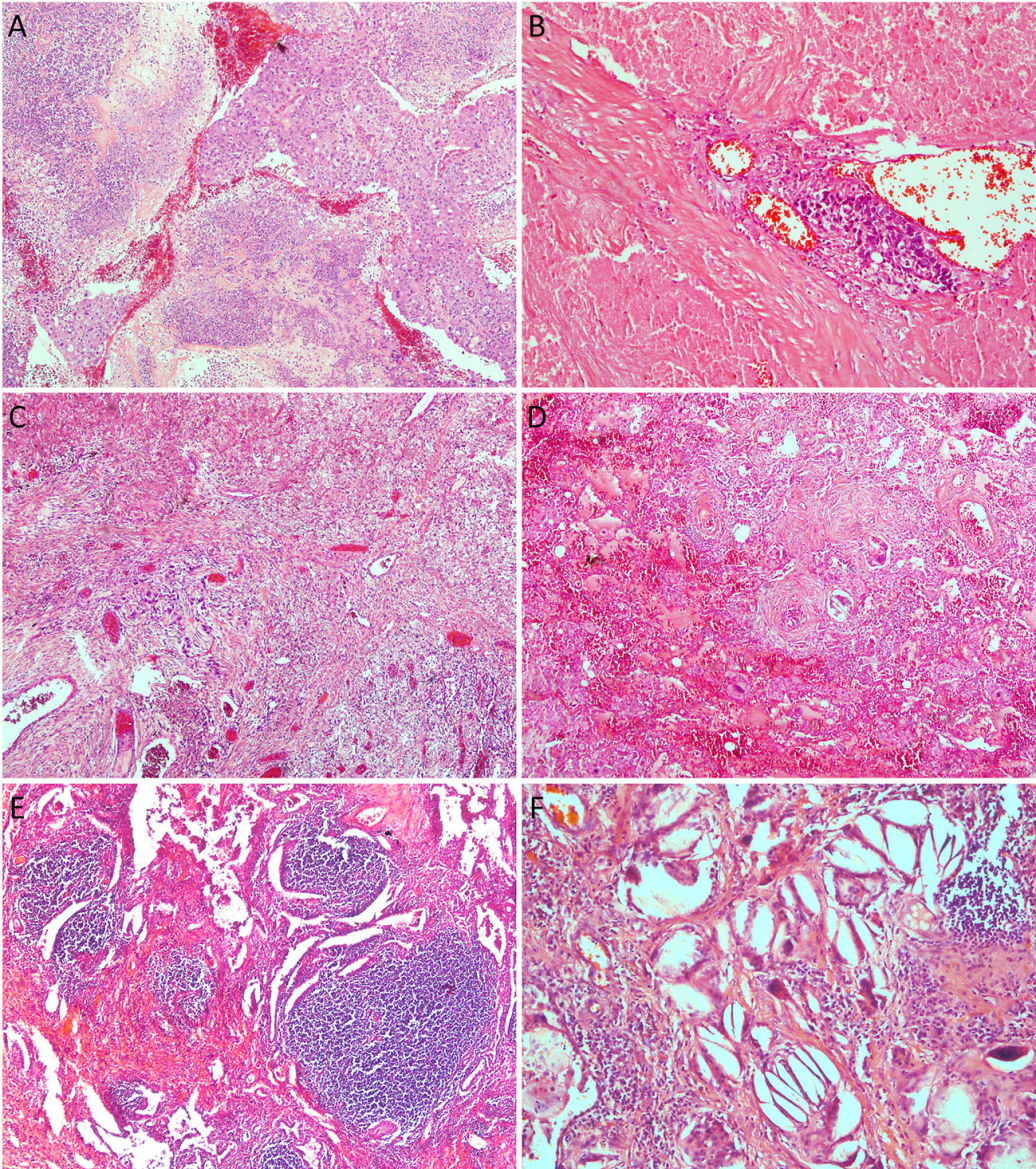
Histologic features after neoadjuvant therapy: **(A)**, area of solid nests of chemotherapy treated squamous cell carcinoma surrounded by necrotic areas (magnification x 10); **(B)**, chemo-immunotherapy treated tumor with a large area of necrosis showing a single focus of viable adenocarcinoma (magnification x 20); **(C)**, focus of atypical cells of chemo-immunotherapy treated tumor with adenocarcinoma histology surrounded by dense fibrosis (magnification x 4); **(D)**, chemotherapy treated tumor with larger area of dense fibrosis with abundant foamy histiocytes (magnification x 4); **(E)**, this area of chemo-immunotherapy treated tumor shows fibrosis with extensive inflammatory infiltrate of lymphocytes and plasma cells (magnification x 4); **(F)**, high power of the same tumor showing chronic inflammation, cholesterol clefts, foamy histiocytes, and fibrosis.

To further explore the pathological response after treatment, we evaluated also several histological features on tumor specimens and in the surrounding lung parenchyma (**Figures 3A–F**). We observed a significantly higher amount of stroma in the neoplastic bed in the cohort of patients treated with immuno-chemotherapy (*p* = 0.004) (**Figure 1**). The evaluation of stroma included fibrosis and inflammation in the tumor bed. However, we did not observe statistically significant differences in terms of fibrosis and inflammation as well as the presence of organizing pneumonia, granuloma, cholesterol cleft, and reactive epithelial alterations. Although these morphological characteristics did not reach statistical significance in the two different treatment cohorts, they were less prominent or absent in patients treated with chemotherapy alone. The complete list of histological features is summarized in **Table 2**.

**Table 2:** Morphological characteristics of tumors after neoadjvant treatment.

Morphological Features	All Patients (N = 26)	Treated CIT Patients (N = 12)	Treated CT Patients (N = 14)	P value
*Necrosis, median (IQR)*	20 (11.5-40)	29 (16.75-42.25)	16 (9.5-28.25)	0.28
*Stroma (fibrosis and inflammation)*, *median (IQR)*	39 (20-56.5)	20 (10-37.25)	55 (47.5-60)	**0.004**
*Tumor inflammation, n (%)* *Yes* *No*	17 (65.4) 9 (34.6)	10 (83.3) 2 (16.7)	7 (50.0) 7 (50.0)	0.11
*Tumor fibrosis, n (%)* *Yes* *No*	19 (73.1) 7 (26.9)	11 (91.7) 1 (8.3)	8 (57.1) 6 (42.9)	0.08
*Parenchyma inflammation, n (%)* *Yes* *No*	15 (57.7) 11 (42.3)	5 (41.7) 7 (58.3)	10 (71.4) 4 (28.6)	0.23
*Parenchyma fibrosis, n (%)* *Yes* *No*	18 (69.2) 8 (30.8)	10 (83.3) 2 (16.7)	8 (57.1) 6 (42.9)	0.22
*Organizing pneumonia, n (%)* *Yes* *No*	18 (69.2) 8 (30.8)	9 (75) 3 (25)	9 (64.3) 5 (35.7)	0.68
*Cholesterol cleft, granuloma, n (%)* *Yes* *No*	17 (65.4) 9 (34.6)	10 (83.3) 2 (16.7)	7 (50.0) 7 (50.0)	0.11
*Reactive epithelial alterations, n (%)* *Yes* *No*	15 (57.7) 11 (42.3)	9 (75) 3 (25)	6 (42.9) 8 (57.1)	0.13

CIT, chemo-immunotherapy; CT, chemotherapy; bold p-value: value below 0.05 was considered significant.

**Figure 4:**
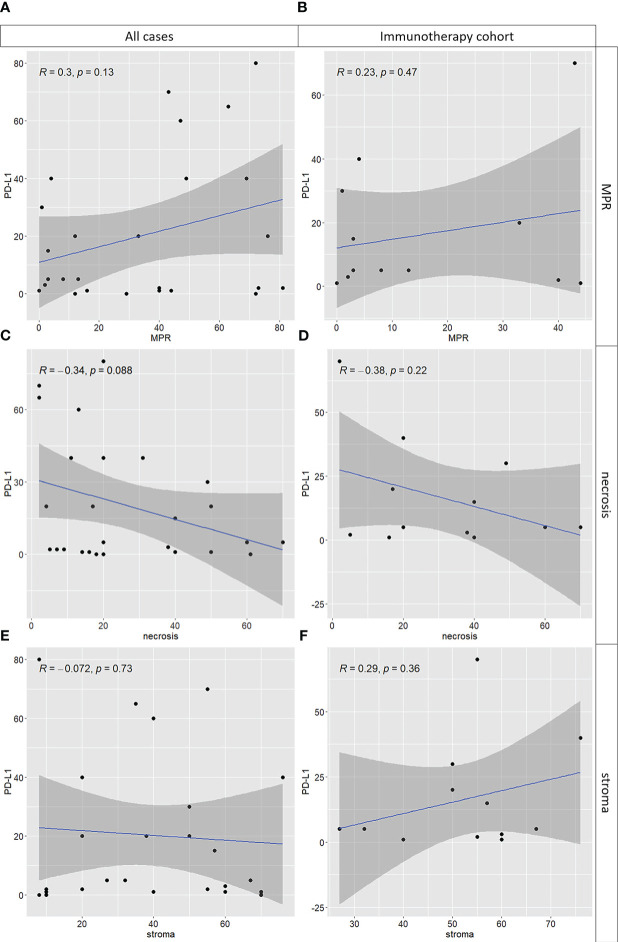
Correlations between PD-L1 tumor proportion score in naïve tumor and tumor components after neoadjuvant treatment including major pathological response (MPR), necrosis and stroma (i.e, inflammatory cells and fibrosis). On the left (i.e., panels **A**, **C**, **E**) the entire cohort of cases was used; on the right (i.e., panels **B**, **D**, **F**) only cases treated with chemo-immunotherapy were used. While for MPR **(A, B)** and necrosis **(C, D)** the results are consistent, a mild positive correlation between PD-L1 levels and stroma is observed in the chemo-immunotherapy cohort only **(F)**, not in the entire cohort **(E)**.

Although no significant correlations were found between PD-L1 immunohistochemical expression and MPR, necrosis, and stroma, we observed a trend showing that PD-L1 levels positively correlated with MPR both in the general cohort and in patients treated with combination therapy. On the contrary, the amount of stroma positively correlated with PD-L1 expression levels only in the combined treated patients (**Figures 4A–F**).

### 3.3 Survival analyses

The last follow-up was in June 2022. For all patients, the overall median follow-up was 23 months (interquartile range, IQR, 16 – 32 months). In the chemotherapy treated patient cohort, the median follow-up was 16 months (IQR, 16 – 26). In the combined immuno-chemotherapy cohort, the median follow-up was 29 months (IQR, 22 – 35 months).

**Figure 5:**
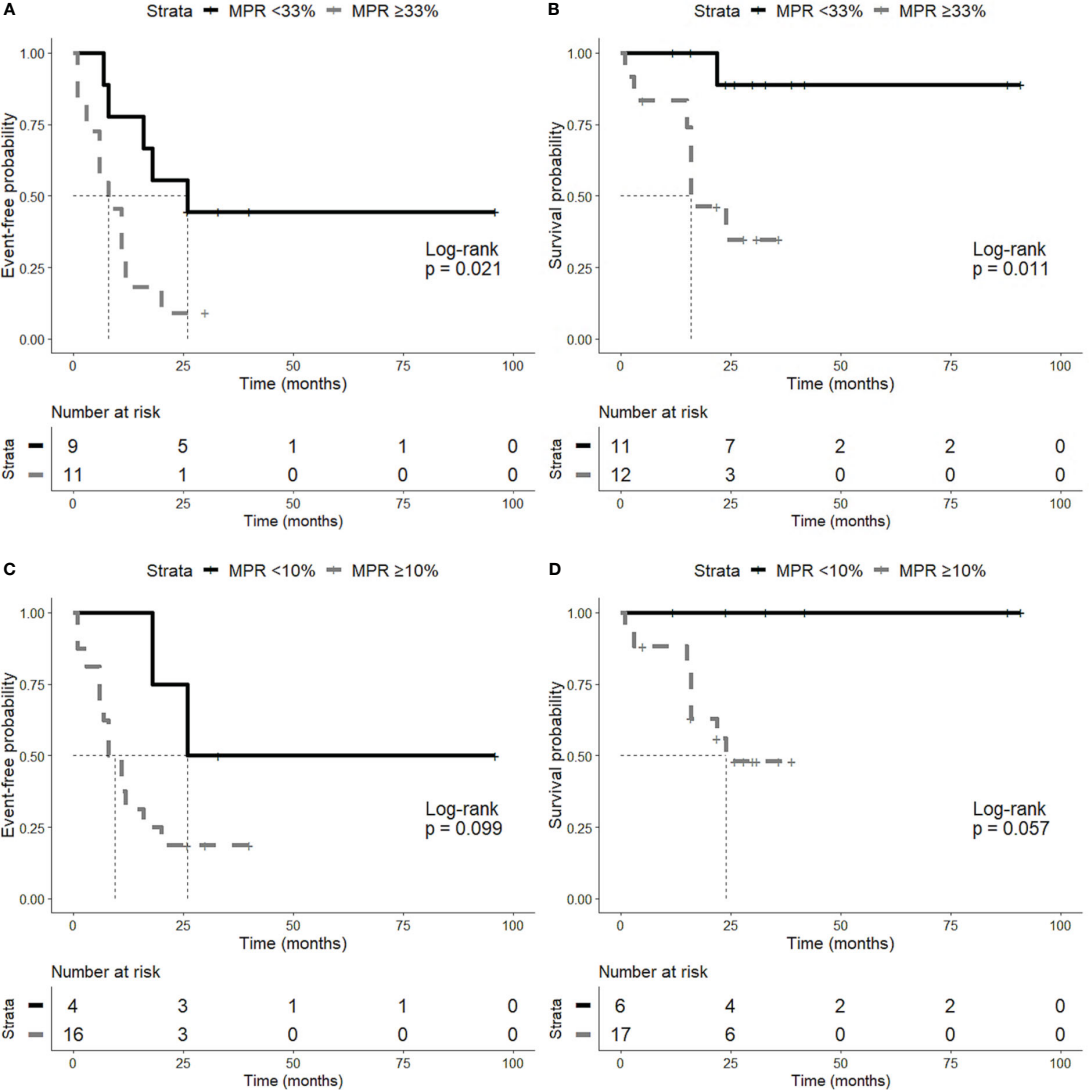
Time-to-event analyses. Patients were stratified according to the best cut-off of major pathological response (MPR) (i.e., 33% of viable tumor cells). Patients with less than 33% of viable tumor cells showed a better event-free survival (EFS) **(A)** and overall survival (OS) **(B)**. The classic 10% MPR cut-off was also tested and showed the same trend both on EFS **(C)** and OS **(D)**.

In univariate analysis and considering the entire cohort of patients, MPR was predictive of long-term OS (*p* = 0.04) and EFS (*p* = 0.04) after neoadjuvant therapy. In particular the best cut-off was 33% of viable tumor cells, which significantly stratified patients according to EFS (*p* = 0.02), and OS (*p* = 0.01) (**Figures 5A–D**).

Regarding histopathological features of tumors, the amount of necrosis was associated with a longer EFS (*p* = 0,02), whereas no association was observed between necrosis and OS.

We observed also a trend for a better overall survival of patients treated with chemo-immunotherapy (*p* = 0.07) and, as expected, a trend for a better outcome of patients with stage I or II disease both in EFS (*p* = 0.12) and OS (*p* = 0.12).

Finally, we performed a multivariate analysis including MPR, stage and treatment. While higher disease stage was predictive of poor EFS (*p* = 0.02) and OS (*p* = 0.05), MPR was associated with EFS independently of stage and treatment regimen (*p* = 0.02) (**Table 3**).

**Table 3:** Univariate and multivariate time-to-event analyses.

	OS		EFS	
	*HR (95% CI)*	*P-value*	*HR (95% CI)*	*P-value*
Univariate Analysis				
MPR* Necrosis Stroma	1.03 (1.00-1.05)	**0.04**	1.02 (1.00-1.04)	**0.04**
	0.97 (0.93-1.01)	0.16	0.96 (0.93-0.99)	**0.02**
	0.97 (0.93-1.01)	0.18	1 (0.97-1.02)	0.84
Stage				
I-II III-IV	1	0.12	1	0.12
	3.09 (0.76-12.59)		2.31 (0.81-6.60)	
Treatment				
CT CIT	1	0.07	1	0.36
	0.14 (0.02-1.19)		0.61 (0.22-1.74)	
Multivariate Analysis				
MPR*	1.02 (0.99-1.05)	0.27	1.03 (1.00-1.06)	**0.02**
Stage				
I-II III-IV	1	**0.05**	1	**0.02**
	4.56 (1.02-20.39)		4.10 (1.20-13.99)	
Treatment				
CT CIT	1	0.20	1	0.94
	0.20 (0.02-2.31)		1.05 (0.28-3.94)	

MPR, major pathological response; MPR*: MPR was used as continuous variable; OS, overall survival; EFS, event free survival; CIT, chemo-immunotherapy; CT, chemotherapy.

Patients were stratified according to MPR, stage and treatment to evaluate differences in OS and EFS.

Bold *p*-value: value below 0.05 was considered significant.

### 3.4 Gene expression analysis

**Figure 6:**
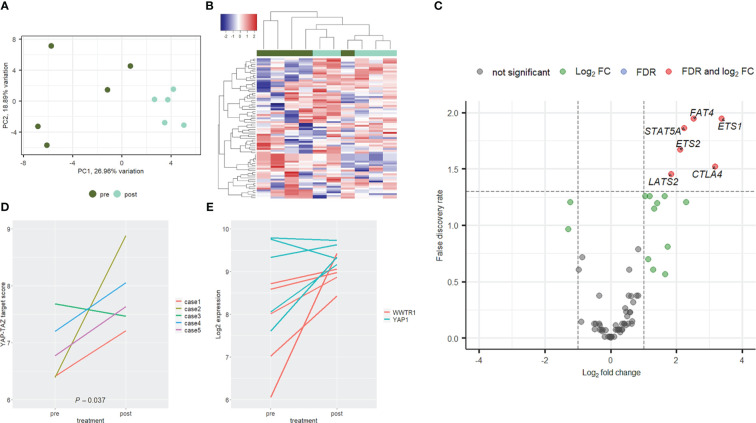
Expression profile of Hippo genes. Paired naïve (green) and post-surgical (cyan) tumors from the chemo-immunotherapy cohort clearly separated at PCA **(A)** and hierarchical clustering **(B)** analyses. In post-surgical specimens a trend towards gene upregulation was observed **(C)**, with six genes significantly deregulated (red dots). YAP-TAZ target score was significantly upregulated in post-surgical samples **(D)**. This was consistent with a trend towards upregulation of genes encoding for YAP and TAZ (i.e., YAP1 and WWTR1 respectively, especially the latter) **(E)**.

Gene expression analysis was performed in a small set of samples, namely 5 tumors from patients treated with neoadjuvant chemo-immunotherapy. For each case, both pre- and post-surgical tissue samples were analyzed to observe changes of Hippo gene expression. Differences between pre- and post-surgical samples were remarkable at unsupervised analyses. In fact, pre- and post-surgical samples were clearly separated at PCA (**Figure 6A**) and heatmap (**Figure 6B**), with only one exception in the latter. Six genes were significantly upregulated in post-surgical samples (**Figure 6C**), namely *ETS1*, *FAT4*, *STAT5A*, *ETS2*, *CTLA4* and *LATS2*. In **Table 4** are reported genes with an FDR below 0.15.

**Table 4:** Genes deregulated after adjuvant chemo-immunotherapy.

Gene symbol	Gene name	Log2 FC	FDR
ETS1	ETS proto-oncogene 1, transcription factor	3.38	0.0113
FAT4	FAT atypical cadherin 4	2.52	0.0113
STAT5A	signal transducer and activator of transcription 5A	2.24	0.0137
ETS2	ETS proto-oncogene 2, transcription factor	2.11	0.0214
CTLA4	cytotoxic T-lymphocyte associated protein 4	3.17	0.0303
LATS2	large tumor suppressor kinase 2	1.84	0.0351
DCHS1	dachsous cadherin-related 1	1.65	0.0552
YWHAB	tyrosine 3-monooxygenase/tryptophan 5-monooxygenase activation protein beta	1.06	0.0552
SMAD7	SMAD family member 7	1.19	0.0552
TEAD1	TEA domain transcription factor 1	2.29	0.0623
AJUBA	ajuba LIM protein	-1.22	0.0623
MYC	MYC proto-oncogene, bHLH transcription factor	1.41	0.0638
RASSF5	Ras association domain family member 5	1.32	0.0715
SCRIB	scribble planar cell polarity protein	-1.29	0.1080

FC, fold change; FDR, false discovery rate.

To evaluate the activation of YAP and TAZ (encoded by *YAP1* and *WWTR1* genes respectively) two approaches were used. First, a YAP-TAZ target score was built by averaging the expression level of 3 validated targets (i.e., *AMOTL2*, *LATS2* and *PTPN14*, DOI: 10.1016/j.celrep.2018.10.001). Second, the mRNA expression of *YAP1* and *WWTR1* were evaluated. As presented in **Figure 6D**, YAP-TAZ target score was always higher in post-surgical samples with one exception. These results were confirmed by the pre- and post-surgical levels of *YAP1* and *WWTR1*. In fact, after neoadjuvant immunotherapy, both genes were upregulated, especially *WWTR1* (**Figure 6E**).

## 4 Discussion

In resectable NSCLC preoperative treatments, including immunotherapy, can improve clinical outcomes and patients survival (6).

To date, there are limited data establishing the prognostic relationship between pathological response after neoadjuvant therapy in resectable NSCLC and clinical outcome, making it an interesting research area. Pathological response has been proposed as a surrogate indicator of benefit to neoadjuvant therapy in order to evaluate the effectiveness of agents tested in clinical trial (18). Numerous studies showed that neoadjuvant chemotherapy treated patients with lung cancer that achieve a MPR ≤10% have a significantly improved survival (10, 12, 19). Therefore, pathological response, including pCR and MPR, can be relevant to assess the impact of neoadjuvant chemo-immunotherapy (10).

Studies evaluating the effect of neoadjuvant immunotherapy in resectable NSCLC have shown a median pathological response of 50-92.5%. In particular, a MPR defined as 10% or less of residual viable tumor cells in the resected primary tumor was reported in 40.5-56.7% of cases, while cPR, defined as no viable tumor within the resected specimen, was reported in 15-33% and 8.1-10% for primary lesions and lymph nodes respectively. These responses are better than those reported for patients treated with neoadjuvant chemotherapy (5, 13, 20–23).

In the present study, we retrospectively analyzed 26 NSCLC patients treated with neoadjuvant therapy of which 14 with chemotherapy alone and 12 with chemo-immunotherapy. In agreement with literature data (5, 13, 20–23), we observed a better pathological response in the chemo-immunotherapy cohort: six patients (50.0%) achieved a MPR and one patient a pCR both on primary tumor and on lymph nodes (8.3%). On the contrary, no patient treated with chemotherapy alone achieved pCR or MPR.

In comparison to our results, Shi L and collaborators reported a higher pathological response in squamous cell lung carcinoma treated with neoadjuvant chemo-immunotherapy (24), with 66.7% of patients achieving MPR and 39.7% cases achieving a pCR. This discrepancy could be due to the different type of specimens included in the study. In fact, they analyzed only squamous histology, whereas we analyzed also adenocarcinoma, large cell neuroendocrine carcinoma, and adenosquamous carcinoma. Previous data showed that squamous cell carcinoma demonstrates a different response to immunotherapy in comparison to non-squamous cell carcinoma, with much more infiltration of immune cells and higher expression of PD-L1 (25–27). Even after neoadjuvant chemotherapy, squamous cell carcinoma shows a greater MPR than adenocarcinoma (28).

Recently, new clinical trials showed an improved EFS in patients treated with neoadjuvant chemo-immunotherapy or immunotherapy alone, as compared to chemotherapy treated patients (5, 6, 29). CheckMate-816 clinical trial compared neoadjuvant nivolumab plus chemotherapy versus neoadjuvant chemotherapy showing longer EFS in patients who achieved pathological response (5). In the present study, we observed a significant association between MPR after neoadjuvant treatment and prognosis. In particular, we observed an association between MPR and both EFS (*p* = 0.04) and OS (*p* = 0.04). These findings were also confirmed by multivariate analysis showing that MPR was associated with EFS independently of stage and treatment regimen (*p* = 0.02). We observed also a trend for a better overall survival of patients treated with chemo-immunotherapy (*p* = 0.07) and, as expected, a trend for a better outcome of patients with stage I and II disease both in EFS (*p* = 0.12) and OS (*p* = 0.12). However, these results did not reach statistical significance probably due to the small number of cases.

Although promising, our results show that a considerable percentage of neoadjuvant chemo-immunotherapy treated patients (40%–75%) still do not achieve MPR or pCR, presenting a higher risk of relapse (5, 23, 30). Thus, the identification of predictive biomarkers of pathological response in resectable NSCLC is needed.

Cottrell and collaborators suggested that immunotherapy responsive tumors showed specific histological changes reflecting a state of immune activation (31). This finding could explain lack of correlation between pathological and radiological responses reported after neoadjuvant immunotherapy, related to the increased size of tumor on imaging caused by the infiltration of T-cells and macrophages, neovascularization and fibrosis (32).

In our study, we evaluated other histological features of tumor bed including necrosis and stroma. Regarding the amount of necrosis, we didn’t observe any significant difference between the two treatment cohorts, whereas we observed a significantly higher amount of stroma in the neoplastic bed in the immuno-chemotherapy treated patients. This observation agrees with previous studies showing a greater amount of fibrosis in patients treated with chemo plus immunotherapy compared to patients treated with chemotherapy alone (18).

Regarding the identification of predictive factors for neoadjuvant treatments, we evaluated immunohistochemical PD-L1 expression since it is a critical marker to guide patient selection for immunotherapy in advanced NSCLC. However, we did not find associations between PD-L1 expression and pathological response or other histological features. We observed only a trend showing that PD-L1 levels positively correlated with MPR both in the general cohort and with MPR and stroma in patients treated with combination therapy. Although some studies reported a greater benefit from the combined chemo-immunotherapy in patients with a high PD-L1 immunohistochemical expression (33, 34), others suggested a lack of correlation between PD-L1 expression of pre-treatment specimens and patients’ response (24, 30). Therefore, PD-L1 expression should not be considered a good predictive marker for neoadjuvant chemo-immunotherapy. Probably an optimal approach should not be based on the analysis of a single marker, but it should be more comprehensive evaluating not only the tumor but also its microenvironment.

In this study, after neoadjuvant chemo-immunotherapy, residuals tumors showed the upregulation of *YAP1* and *WWTR1*, which encode for YAP and TAZ respectively. Consistently, YAP/TAZ target expression was enhanced. These findings are consistent with high levels of fibrosis in these tumors since the activation of YAP/TAZ is crucial in regulating tissue repair (doi: 10.1038/s41573-020-0070-z). However, this activation could suggest also the selection of cells resistant to treatment (doi: 10.1016/j.cell.2014.06.004), and could open new perspectives in further lines of treatment. Similarly, the enhanced expression of *CTLA4* after treatment with PD-1 or PD-L1 agonists, could be a resistance mechanism that should be considered after progression to PD-1/PD-L1 blockade.

Several limitations associated with the present study should be mentioned. First, the small sample size made it difficult to obtain robust statistical results and a further validation is warranted. Second, this was a retrospective, non-randomized single-center study needing to be confirmed in prospective cohorts. Moreover, our study lacks long-term follow up that will be necessary to evaluate the efficacy of neoadjuvant chemo-immunotherapy on recurrence and survival in resectable NSCLC.

In conclusion, despite these limitations, our study demonstrated that the combination of immunotherapy and chemotherapy in neoadjuvant setting significantly improves pathological response in comparison to chemotherapy alone. At the same time, we suggested that chemo-immunotherapy could induce different morphological and molecular changes of treated specimens, both of the tumor and of the collateral lung parenchyma, in comparison to chemotherapy alone. These differences can impact on specimens processing and scoring in the evaluation of pathological response, and can increase our knowledge of biological and histological features of responders and non-responders to different neoadjuvant therapies.

## Data availability statement

The datasets presented in this study can be found in online repositories. The names of the repository/repositories and accession number(s) can be found in the article/**Supplementary Material**.

## Ethics statement

The studies involving human participants were reviewed and approved by “Comitato Etico di Area Vasta Nord Ovest” (CEAVNO) for Clinical Experimentation (Protocol Number: ID19211). The patients/participants provided their written informed consent to participate in this study.

## Author contributions

GA, EB, FM, and GF contributed to conception and design of the study. AL, AC, and GM organized the database. AP performed the statistical analysis. GA and IS wrote the first draft of the manuscript. GA, GR, and CZ wrote sections of the manuscript. All authors contributed to manuscript revision, read, and approved the submitted version.
